# Supplementary material for: Identification and characterization of early human photoreceptor states and cell-state-specific retinoblastoma-related features
Source: eLife. 2025 Aug 6;13:RP101918. doi: 10.7554/eLife.101918 (PMC12327943; doi:10.7554/eLife.101918)
Supplement: Figure 3—figure supplement 2—source data 1. [file elife-101918-fig3-figsupp2-data1.zip › Shayler Figure 3-figure supplement 2-source data 1 pdf/Figure 3-figure supplement 2-source data.pdf]

Figure 3-figure supplement 2a (*left*)

Goat anti-total NRL

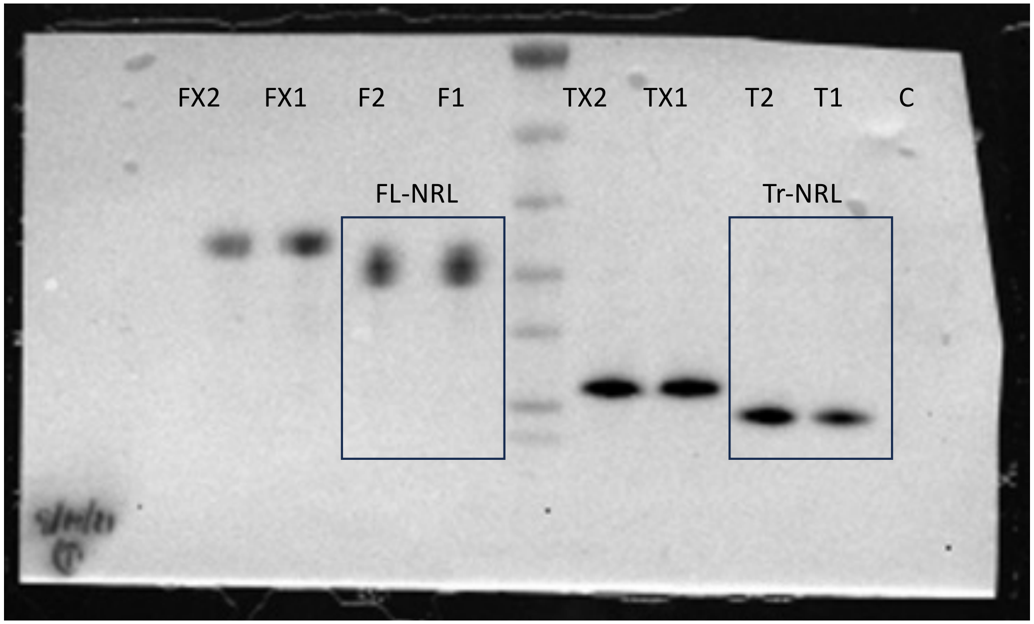

Figure 3-figure supplement 2a (*right*)

Mouse anti-N-term NRL

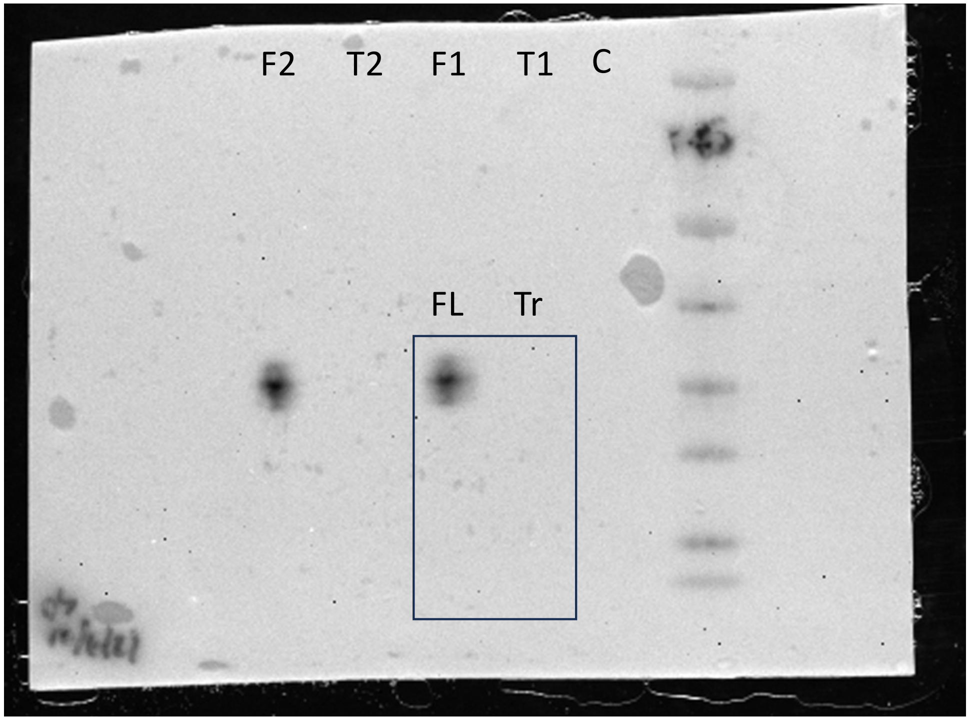

Figure 3-figure supplement 2c

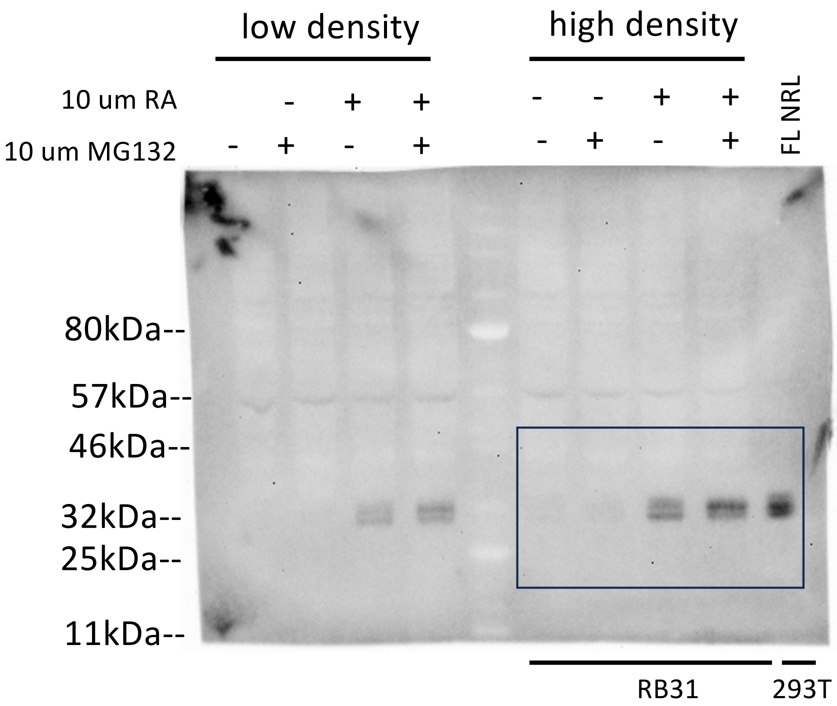

-
