# Supplementary figures and images for: Identification and characterization of early human photoreceptor states and cell-state-specific retinoblastoma-related features
Source: eLife. 2025 Aug 6;13:RP101918. doi: 10.7554/eLife.101918 (PMC12327943; doi:10.7554/eLife.101918)

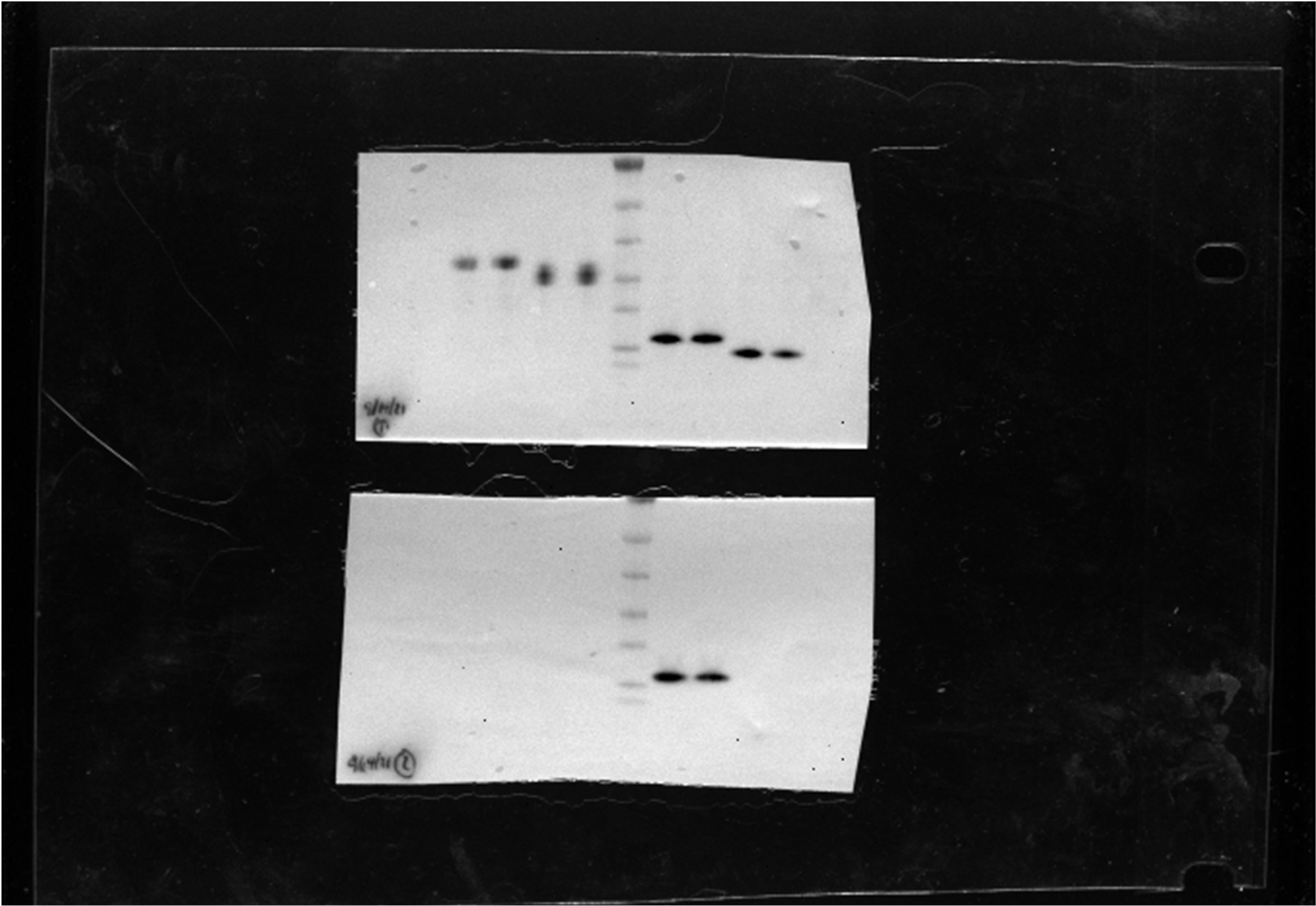

Supplement: Figure 3—figure supplement 2—source data 2. [file elife-101918-fig3-figsupp2-data2.zip › Shayler Figure 3-figure supplement 2-source data 2/Figure 3-figure supplement 2A-left source data.jpg]

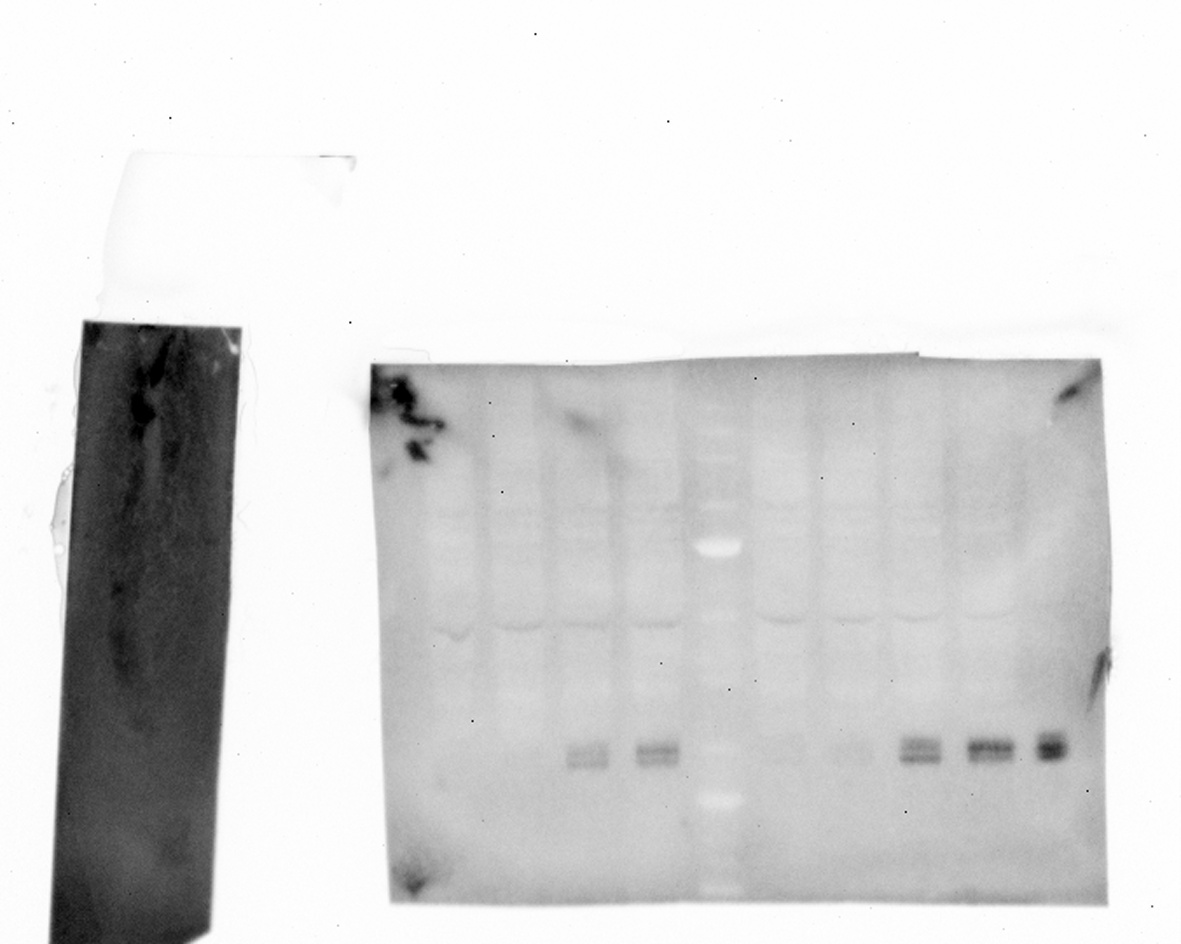

Supplement: Figure 3—figure supplement 2—source data 2. [file elife-101918-fig3-figsupp2-data2.zip › Shayler Figure 3-figure supplement 2-source data 2/Figure 3-Figure Supplement 2C source data.jpg]

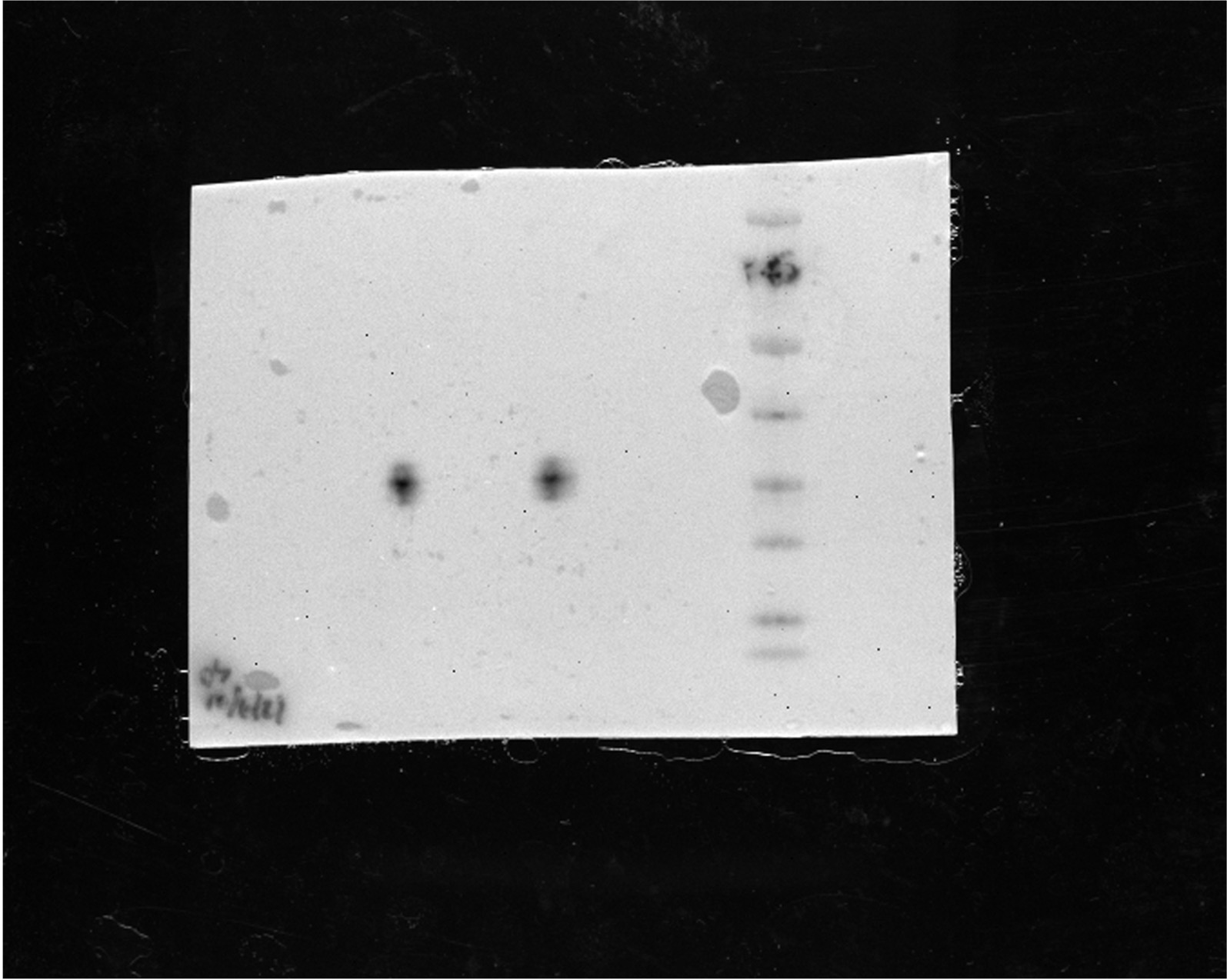

Supplement: Figure 3—figure supplement 2—source data 2. [file elife-101918-fig3-figsupp2-data2.zip › Shayler Figure 3-figure supplement 2-source data 2/Figure 3-figure supplement 2A-right source data.jpg]
